# Gut microbiome dynamics in index patients colonized with extended-spectrum beta-lactamase (ESBL)-producing Enterobacterales after hospital discharge and their household contacts

**DOI:** 10.1128/spectrum.01275-23

**Published:** 2023-10-27

**Authors:** Janetta Top, Tess D. Verschuuren, Marco C. Viveen, M. Eugenia Riccio, Stephan Harbarth, Jan A. J. W. Kluytmans, Rob J. L. Willems, Fernanda L. Paganelli

**Affiliations:** 1 Department of Medical Microbiology, University Medical Center Utrecht, Utrecht, the Netherlands; 2 Mahidol Oxford Tropical Medicine Research Unit (MORU), Bangkok, Thailand; 3 University of Geneva Hospitals and Faculty of Medicine, Infection Control Program, WHO Collaborating Center, Geneva, Switzerland; University of Arkansas for Medical Sciences, Little Rock, Arkansas, USA

**Keywords:** ESBL-Enterobacterales, gut microbiome dynamics, index patient, household contacts

## Abstract

This study investigated the gut microbiome dynamics of index patients colonized with extended-spectrum beta-lactamase-producing *Escherichia coli* (ESBL-Ec) (*n* = 5) or extended-spectrum beta-lactamase-producing *Klebsiella pneumoniae* (ESBL-Kp) (*n* = 3) and their household contacts (*n* = 9) up to 4 months after hospital discharge of the index patient using 16S rRNA amplicon gene sequencing. Samples were collected at the day of hospital discharge of the index patient, 1 week and 2 and 4 months after discharge. Compared to household contacts, we observed a significant lower alpha diversity (*P* < 0.001) among index patients and significant (*P* < 0.05) separation between the two groups for beta diversity. Principal component analysis of the samples from each household (i.e., index patient and respective household contact) showed a clear shift in microbiome composition, in 4/8 index patients, from dissimilar to more similar to the household contact group. This suggests recovery of the microbiome to a healthier status, which was also reflected by *de novo* colonization of (health-associated) taxa. In contrast, the four time-point samples of the household contacts clustered together indicating a stable microbiome composition over time irrespective of low-level ESBL-Ec (*n* = 3) or ESBL-Kp (*n* = 2) colonization. In conclusion, here we show that, at the day of hospital discharge, the microbiome composition of index patients is dissimilar from that of household contacts. Over time, signals of microbiome recovery were observed in half of the index patients. The stable microbiome composition in household contacts irrespective of low-level ESBL-Ec or ESBL-Kp colonization suggests that the gut microbiome in these healthy people provided colonization resistance against ESBL-PE outgrowth.

**IMPORTANCE:**

Colonization with extended-spectrum beta-lactamase-producing Enterobacterales (ESBL-PE) often precedes infections and is therefore considered as a great threat for public health. Here, we studied the gut microbiome dynamics in eight index patients colonized with ESBL-PE after hospital discharge and the impact of exposure to this index patient on the gut microbiome dynamics of their household contacts. We showed that the microbiome composition from index patients is different from their household contacts upon hospital discharge and that, in some of the index patients, their microbiome composition over time shifted toward the composition of their household contacts. In contrast, household contacts showed a stable microbiome composition over time irrespective of low-level extended-spectrum beta-lactamase-producing *Escherichia coli* (ESBL-Ec) or extended-spectrum beta-lactamase-producing *Klebsiella pneumoniae* (ESBL-Kp) gut colonization, suggesting that, in healthy microbiomes, colonization resistance is able to prevent ESBL-PE expansion.

## INTRODUCTION

The transmission of extended-spectrum beta-lactamase-producing Enterobacterales (ESBL-PE) in clinical settings has been extensively studied (1). However, less is known about human-to-human ESBL-PE transmission in the community (2 – 5). In a recent systematic review, which compared the global prevalence and trend of intestinal carriage of extended-spectrum beta-lactamase-producing *Escherichia coli* (ESBL-Ec) between healthcare and community settings, the authors concluded that ESBL-Ec is increasing in both settings and emphasized the need for surveillance and implementation of preventive measures to limit the spread of ESBL-Ec (6). In another systematic review, which investigated ESBL-PE co-carriage, defined as simultaneous carriage of a related ESBL-PE strain by two or more household members at a certain point in time or during a predefined follow-up period, and acquisition in households, it was concluded that co-carriage is frequent, suggesting intra family acquisition (7). Recently, Riccio et al. conducted a prospective multicenter cohort study in five European cities with the aim to determine rates and risk factors for the acquisition and transmission of ESBL-PE within households after hospital discharge of an ESBL-PE colonized index patient (8). In that study, index patients colonized with ESBL-Ec or extended-spectrum beta-lactamase-producing *Klebsiella pneumonia* (ESBL-Kp) and their household contacts were followed for 4 months after hospital discharge of the index patient. This study revealed that index patients colonized with ESBL-PE are an important source of ESBL-PE transmission within households and that most of the acquisition and transmission events occurred during the first 2 months after hospital discharge (8).

None of the aforementioned studies investigated the impact of microbiome composition on ESBL-PE acquisition and colonization or vice versa. Over the last few years, there has been an increased interest in the microbiome composition in relation to ESBL-PE acquisition. Davies et al. performed point-prevalence metagenomics on fecal samples from international travelers pre- and post-travel, observed an altered microbiome composition during traveling, and found that this shift in microbiome composition was mainly associated with the development of travelers’ diarrhea and not with the acquisition of ESBL-Ec (9). In another point-prevalence study, the authors also noticed no differences in diversity parameters or relative abundance on bacterial species level in the gut microbiome between healthy people who were colonized or not colonized with ESBL-PE (10). It is important to note that these two studies only investigated the gut microbiome composition at a single time point.

By contrast, in the present study, we used longitudinally collected fecal samples from index patients recently discharged from the hospital and their household contacts. Samples, collected by the University Medical Center Utrecht, originated from the study by Riccio et al. (8) with the aim to investigate the gut microbiome dynamics of index patients colonized with ESBL-PE and their household contacts exposed to the recently discharged index patients.

## MATERIALS AND METHODS

### Sample collection and DNA extraction

Samples from ESBL-PE-positive index patients and their household contacts from the University Medical Center Utrecht, the Netherlands. were included in this study (8). Characteristics of the participants were available from the questionnaires of the previous study (8). The index patients and household contacts were sampled on the day of hospital discharge of the index patient and 1 week, 2 months, and 4 months after hospital discharge (8). DNA extraction was performed using a modified protocol of the QIAamp fast DNA stool mini kit (Qiagen, Venlo, the Netherlands) as previously described (11, 12). In brief, 0.2 g of feces was added to “lysing matrix A, 2 mL tubes” (MP biomedicals, Landsmeer, the Netherlands) containing 1 mL InhibitEx buffer (Qiagen). Two rounds of bead beating were applied at 3.5 m/s for 2 min, followed by 2 min incubation on ice using the FastPrep-24 (MP biomedicals). After 7 min of incubation at 95°C, the fast DNA stool mini kit protocol (Qiagen) was resumed at the proteinase K treatment step. Total DNA was quantified by Picogreen assay (Thermo Fisher Scientific, Waltham, MA, USA). The 469 bp V3 and V4 hyper-variable regions of the 16S rRNA gene were amplified and sequenced using the Illumina MiSeq instrument and Reagent Kit v3 (600-cycle) according to Fadrosh et al. (13). Negative controls and mock communities [ZymoBIOMICS microbial community standard (D6300) and ZymoBIOMICS microbial community DNA standard (D6305), ZymoBIOMICS Microbial Community Standard II (Log Distribution) (D6310), Zymo research, USA] were used from the beginning of DNA isolation up to the data analysis stage and matched with the distribution expected mock compositions (Table S1).

### Bioinformatics

The QIIME2 microbial community analysis pipeline (version 2021.4) (14) was used with DADA2 for sequence variant detection (with default settings, except for --p-trunc-len-f 255 --p-trunc-len-r 240) (15), and SILVA was used as 16S rRNA reference gene database (SILVA 138) (16). Samples contained on average 31.6 k reads, while the negative controls contained on average 95.8 reads.

### Data analysis and statistical methods

All analyses were performed using R 4.0.3 (2020-10-10) in RStudio (version 1.3.1093) and the functions of the packages phyloseq and ggplot2 (17 – 19). Alpha diversity was calculated using the vegan package (20). Beta diversity was visualized using euclidean distance principal component analysis (PCA) based on central log transformation of the data and principal coordinates analysis (PCoA) on Bray-Curtis dissimilarities using plot ordination functions from the microbiome package (21). Correlations were tested using PERMANOVA with the adonis function at 999 permutations of the vegan package. GraphPad Prism (version 9.3.0) was used to plot Bray-Curtis dissimilarities between index patients and household contacts at each of the four-time points. R was used to calculate pairwise comparisons between time points using the Wilcoxon rank sum test, while the Friedman test was used to test for a trend over time. For the latter, as the Friedman test requires complete data sets from all time points, we excluded all pairwise comparisons with index patients from households 1 and 2, while we lacked the day of discharge sample. In addition, GraphPad Prism was used to plot relative abundances of phyla and genera and to calculate significant differences using Mann-Whitney testing.

### Analysis of health-associated taxa

In order to identify signals of microbiome recovery in the index patients, health-associated taxa as decribed by Gupta et al. and Gacesa et al. (22, 23) were determined for each participant time-point sample. Next, from the health-associated taxa that were found per household, we calculated their prevalence and their total relative abundance per time point for the household contact and index patient.

## RESULTS

### Study set of participants and samples

In total, 66 fecal samples were included, representing households 2, 5, and 13 with an ESBL-Kp colonized index patient and households 1, 6, 8, 9, and 10 with an ESBL-Ec colonized index patient and their household contacts as reported in the previous study (8) (Table 1). In addition, in this study, ESBL-Kp and ESBL-Ec were cultured from the household contacts of households 5 and 13 and households 1, 6, and 10, respectively (8) (Table 1). The index patients and household contacts were sampled on the day of hospital discharge of the index patient and 1 week, 2 months, and 4 months after hospital discharge (Table 1). For household numbers 1 and 2, no sample was available from the index patient at the day of discharge. For household 10, fecal samples were obtained from two household contacts, including the partner and the adult child of the index patient (Table 1). Five of the eight index patients used antibiotics prior to two or more of the sampling time points (Table 1), while none of the household contacts used antibiotics (data not shown). Index patients were hospitalized between 3 and 18 days prior to the day of discharge (Table S2). Seven of eight index patients were diagnosed with a malignancy and one with cardiovascular disease (Table S2). Other comorbidities and medication are depicted in Table S2. All participants kept the same diet, i.e., omnivore. None of the household contacts used other medications.

**TABLE 1:** Overview of antibiotic use and ESBL culture results

		Antibiotic use index patient				Culture results ESBL-producing *Enterobacteriaceae* (ESBL-PE)^*b*^			
Household	Participant	Day of discharge	One week	Two months	Four months	Day of discharge	One week	Two months	Four months
1	Index patient	NA^*a*^	no	Fluoroquinolone	yes, unkonwn	NA	ESBL-Ec	ESBL-Ec	ESBL-Ec
	Household contact					ESBL-Ec	ESBL-Ec	ESBL-Ec	ESBL-Ec
2	Index patient	NA	Phosphonic, cefalosporin, sulfonamide	Penicillin	Penicillin	NA	ESBL-Kp	ESBL-Kp	ESBL-Kp
	Household contact					neg	neg	neg	neg
5	Index patient	no	no	no	no	ESBL-Kp	ESBL-Kp	ESBL-Kp	ESBL-Kp
	Household contact					ESBL-Kp	ESBL-Kp	ESBL-Kp	ESBL-Kp
6	Index patient	Macrolide	Macrolide	Fluoroquinolone	Carbapenem	ESBL-Ec	ESBL-Ec	ESBL-Ec	ESBL-Ec
	Household contact					ESBL-Ec	ESBL-Ec	ESBL-Ec	ESBL-Ec
8	Index patient	no	no	no	no	ESBL-Ec	ESBL-Ec	ESBL-Ec	ESBL-Ec
	Household contact					neg	neg	neg	neg
9	Index patient	no	no	no	no	ESBL-Ec	neg	neg	neg
	Household contact					neg	neg	neg	neg
10	Index patient	Cefalosporin	Cefalosporin	no	no	ESBL-Ec	ESBL-Ec	ESBL-Ec	ESBL-Ec
	Household contact					ESBL-Ec	ESBL-Ec	ESBL-Ec	ESBL-Ec
	Household contact^*c*^					neg	neg	neg	neg
13	Index patient	Cefalosporin, penicillin	Cefalosporin, penicillin	no	Penicillin	ESBL-Kp	ESBL-Kp	No-ESBL	ESBL-Kp
	Household contact					ESBL-Kp	neg	neg	neg

^*a*^ NA: not apllicable, no sample.

^*b*^ ESBL-Ec: ESBL-*E. coli*; ESBL-Kp: ESBL-*K. pneumoniae*; neg: No-ESBL.

^*c*^ For household 10, two household contacts provided samples.

### The microbiome composition of index patients is different from that of household contacts

**Fig 1:**
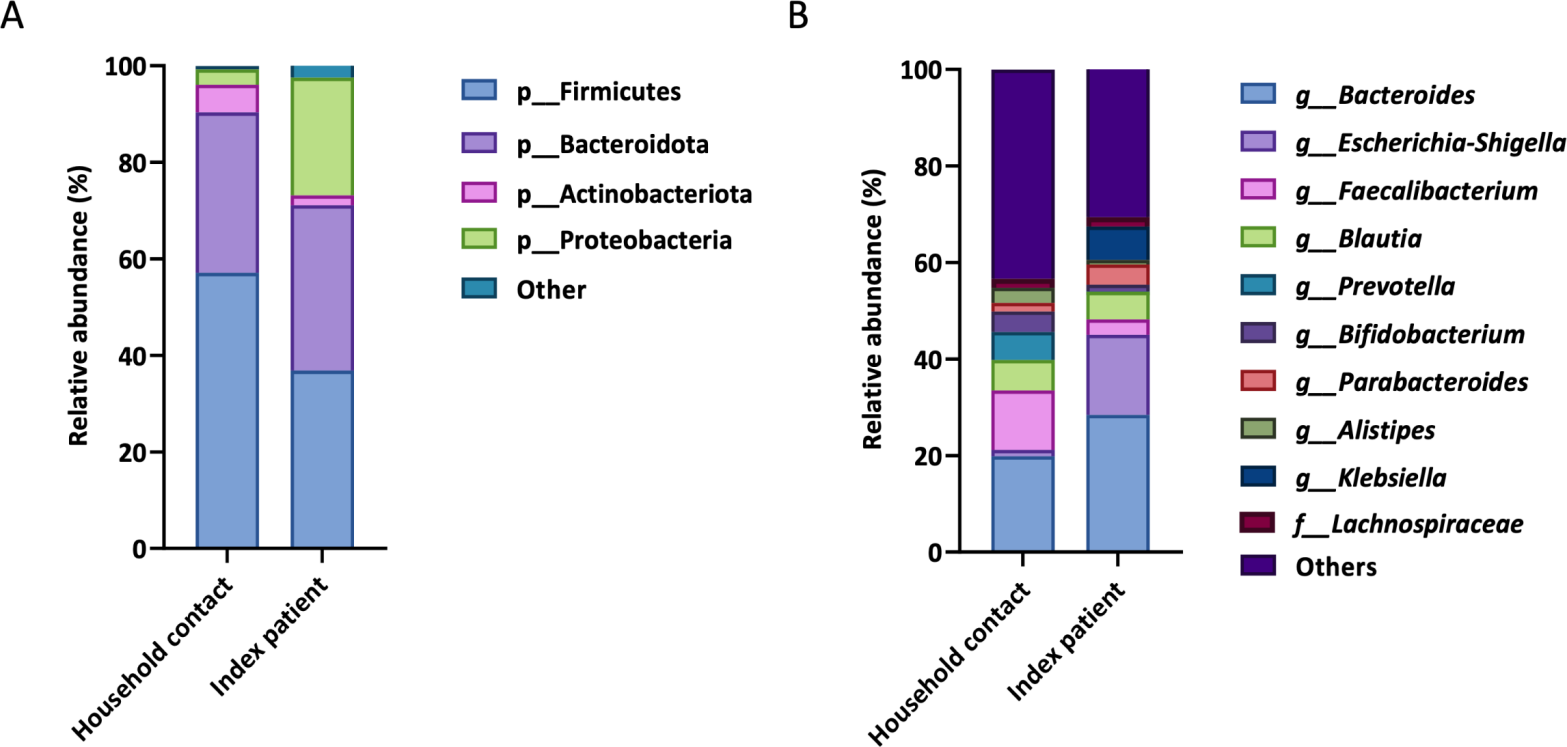
Composition of the fecal microbiota among household contacts and index patients as determined using 16S rRNA gene sequencing. (A) Mean relative abundance top-4 most abundant bacterial phyla. (B) Mean relative abundance top-10 most abundant bacterial genera.

Taxonomic characterization of the fecal microbiome encompassing 272 different amplicon sequence variants revealed significant differences in relative abundance at phylum level between household contacts and index patients for Firmicutes with 57.1% and 36.8% (*P* < 0.0001), Actinobacteriota with 5.8% and 2.1% (*P* < 0.001), and Proteobacteria with 3.2% and 24.4% (*P* < 0.01), respectively (Fig. 1A; Fig. S1). No significant difference was found for the fourth major phylum Bacteroidota with 33.2% and 34.2%, respectively (Fig. 1; Fig. S1). Among the top-10 most abundant genera, *Bacteroides* (*P* < 0.05), *Escherichia-Shigella* (*P* < 0.05), and *Klebsiella* (*P* < 0.01) were significantly more abundant in index patients relative to household contacts, while *Faecalibacterium* (*P* < 0.0001), *Prevotella* (*P* < 0.01), *Bifidobacterium* (*P* < 0.01), and *Alistipes* (*P* < 0.0001) were significantly less abundant in index patients relative to household contacts (Fig. 1B; Fig. S2). Only for *Blautia, Parabacteroides*, and Lachnospiraceae that the difference in relative abundance between index patients and household contacts was not significant (Fig. 1B; Fig. S2).

**Fig 2:**
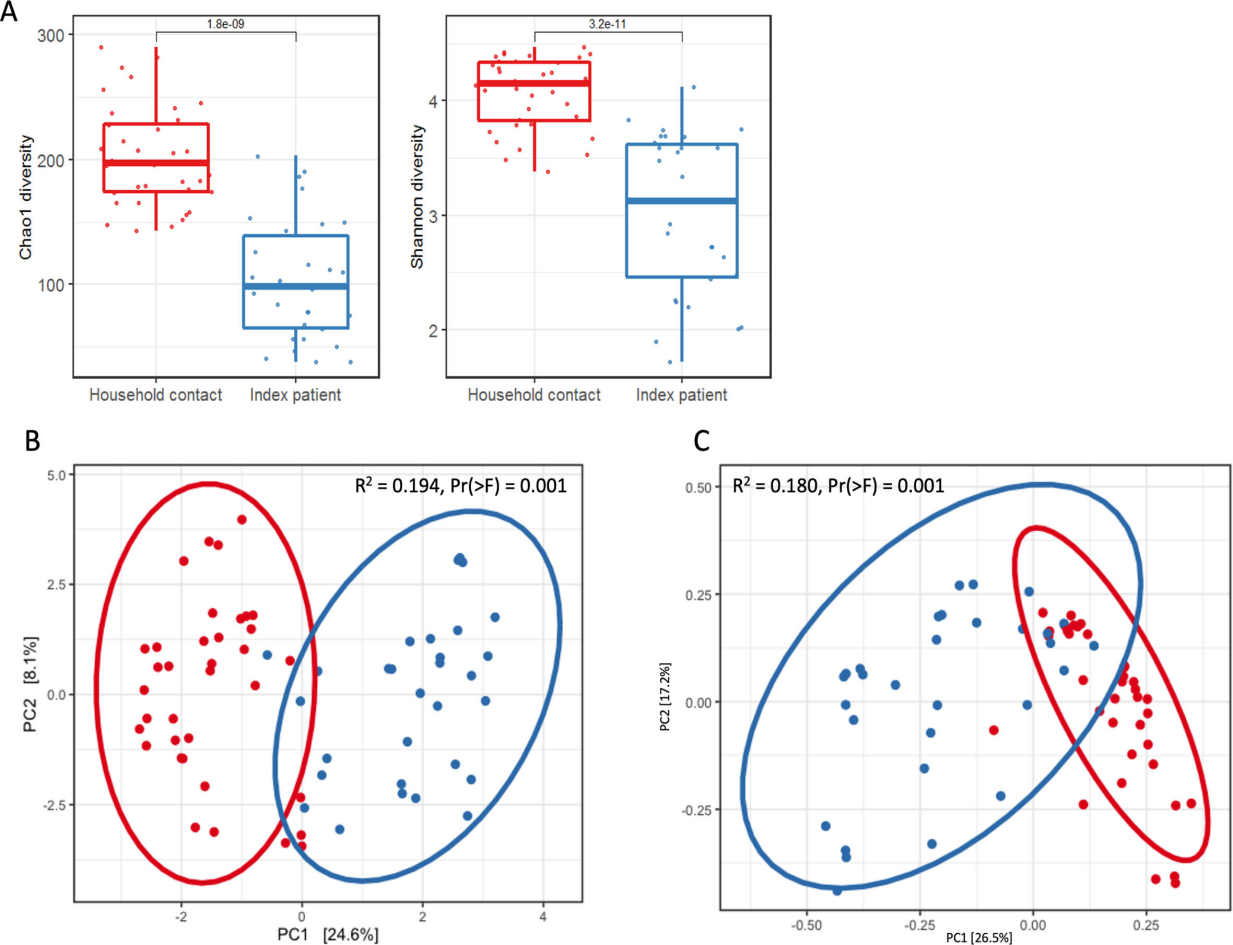
Diversity analysis on fecal samples from household contacts (in red) and index patients (in blue), including all time points. (A) Alpha diversity expressed by Chao1 and Shannon. (B) Euclidean distance PCA plot and (C) Bray-Curtis dissimilarity PCoA plot, both based on bacterial genera and tested with PERMANOVA.

The differences in microbiome composition at phylum and genus level are also reflected in significant lower alpha diversity (*P* < 0.001) among index patients compared to household contacts for Chao1 (richness) and Shannon (both richness and evenness) (Fig. 2A). In addition, genus level beta diversity analysis using PCA based on euclidean distance (Fig. 2B) and PCoA based on Bray-Curtis dissimilarities (Fig. 2C) revealed significant differences [PERMANOVA R^2^ = 0.194, Pr(>F) =0.001 and R^2^ = 0.180, Pr(>F) =0.001, respectively] in overall community composition of the gut microbiome between index patients and their household contacts.

### Analysis of microbiome composition per household

**Fig 3:**
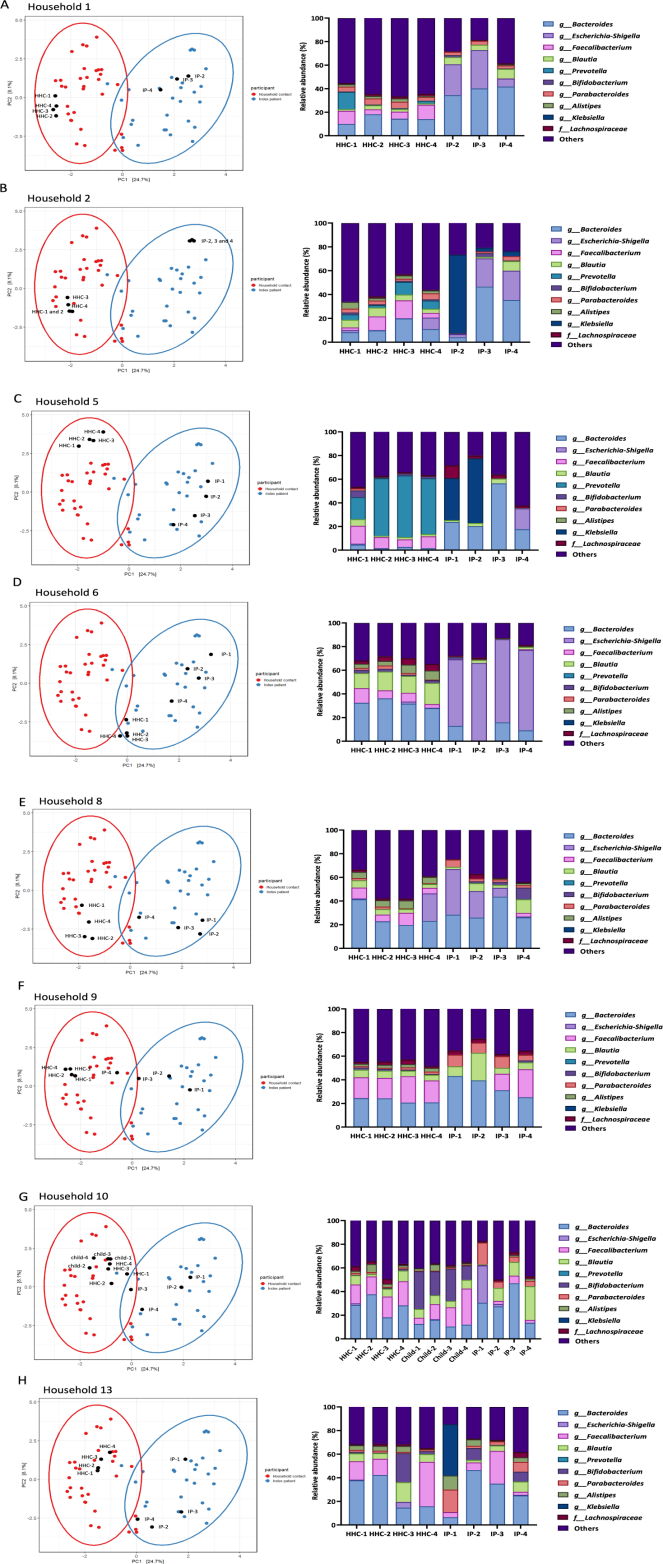
PCA plot and relative abundance of top-10 most abundant taxa for each sample per household. (A) Samples from household number 1. (B) Samples from household number 2. (C) Samples from household number 5. (D) Samples from household number 6. (E) Samples from household number 8. (F) Samples from household number 9. (G) Samples from household number 10. (H) Samples from household number 13. HHC: household contact. IP: index patient.

In order to investigate the dynamics of the microbiome composition over time, we analyzed the relative abundance of the 10 most abundant genera per sample of the index patient and household contacts during all time points per individual household (Fig. 3; Tables S3 through S10). In addition, we marked these samples in the PCA ordination plot displaying overall gut microbiome composition (Fig. 3; Tables S3 through S10). In all individual households, the four samples of household contacts displayed a similar distribution of relative abundances of most abundant genera and clustered together in the PCA ordination plot. This indicates that the gut microbiome composition in household contacts is stable over time. This was also the case for household contacts that were culture positive for ESBL-Ec (household numbers 1, 6, and 10) or ESBL-Kp (household numbers 5 and 13). In these households, the relative abundance of *Escherichia-Shigella* or *Klebsiella* remained very low, suggesting that ESBL-PE gut colonization in these healthy people did not result in intestinal outgrowth of ESBL-PE or other major shifts in the gut microbiome composition over time (Table 1; Tables S3 through S10).

In contrast, the distribution of relative abundances of the 10 most abundant genera in all index patients changed over time. In the PCA ordination plots, all index patient samples from different time points, with the exception of samples from the index patient of household 2, did not group together. This suggests significant changes in the gut microbiome composition of index patients over time (Fig. 3). Although the distribution of the 10 most abundant genera at different time points in samples of index patient 2 also changed, the grouping of these three-time point samples in the PCA plot suggests that the overall gut microbiome composition in this index patient did not change importantly over time.

Detailed analysis of the microbiome composition in samples of index patients taken at the different time points confirmed notable shifts with a decrease in relative abundance of *Escherichia-Shigella* in index patients 1, 8, and 10 and a decrease of relative abundance of *Klebsiella* in index patients 2 and 5 (Tables S3 through S10). In addition, for index patients who received antibiotics, we investigated whether we could observe a general effect of antibiotic-induced microbiota composition as described recently (24). For this analysis, we considered the household contact as healthy control to determine increase or decrease in taxa for index patients within the same household. For each index patient, some general effects were observed, e.g., fluoroquinolone treatment of index patient 1 may have resulted in increase of *Escherichia-Shigella*, probably representing ESBL-Ec, and decrease in Lachnospiraceae and *Coprococcus* (Table S3)*,* while cephalosporin treatment potentially resulted in increased presence of *Bacteroides* in index patients 2 and 10 (Tables S4 and S9), and macrolide treatment of index patient 6 in an increase of *Escherichia-Shigella* and *Enterococcus* (Table S6), while no clear effect was observed for index patient 13 (Table S10)(24).

### Recovery of microbiome in index patients

**Fig 4:**
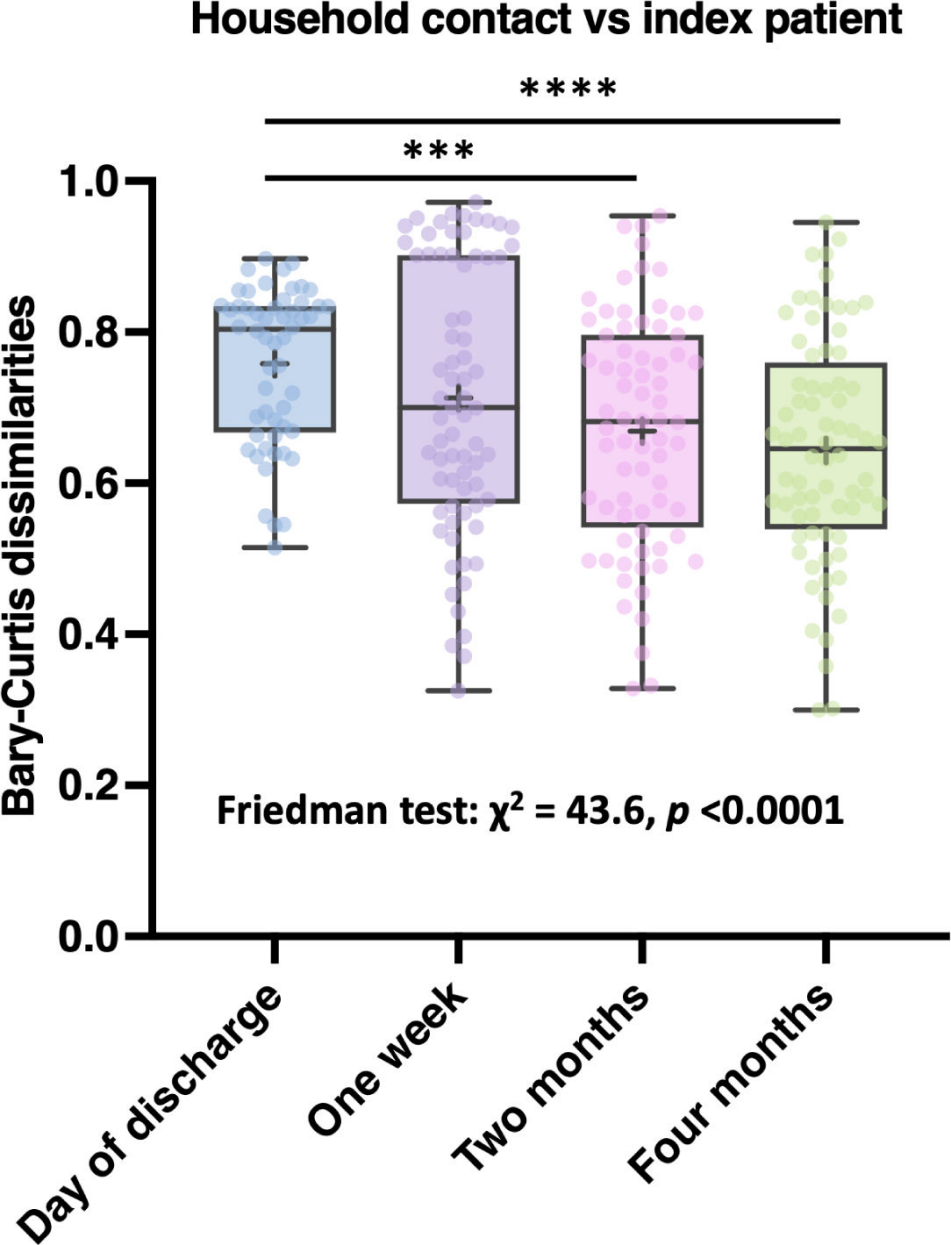
Bray-Curtis dissimilarity between household contacts and index patients per time point. Box and whisker plots represent the mean and min and max values, and the median is represented by an “+”. Wilcoxon rank sum test: ***P < 0.001, ****P < 0.0001. Text beneath the box and whisker plots indicated results of the Friedman trend test.

In order to quantify the observed changes in microbiome composition over time, we calculated Bray-Curtis dissimilarities between household contacts and index patients for each time point (Fig. 4). This analysis revealed an over time decrease in the mean dissimilarity, which was significant between the day of discharge with the 2-month (*P* < 0.001) and 4-month samples (*P* < 0.0001), and a significant trend over time (*P* < 0.0001). This suggests that the microbiome compositions of index patients are shifting from being dissimilar to being more similar to the gut microbiome composition of their household contacts.

Further analysis of the gut microbiome composition of the index patients at different time points revealed that this shift was especially apparent for the index patients from households 8, 9, 10, and 13 (Fig. 3E through H) while it was less apparent in index patients of households 1, 5, and 6 and was absent in household 2 (Fig. 3A through D). This finding suggests recovery of the microbiome in index patients from households 8, 9, 10, and 13 over time after hospital discharge.

**Fig 5:**
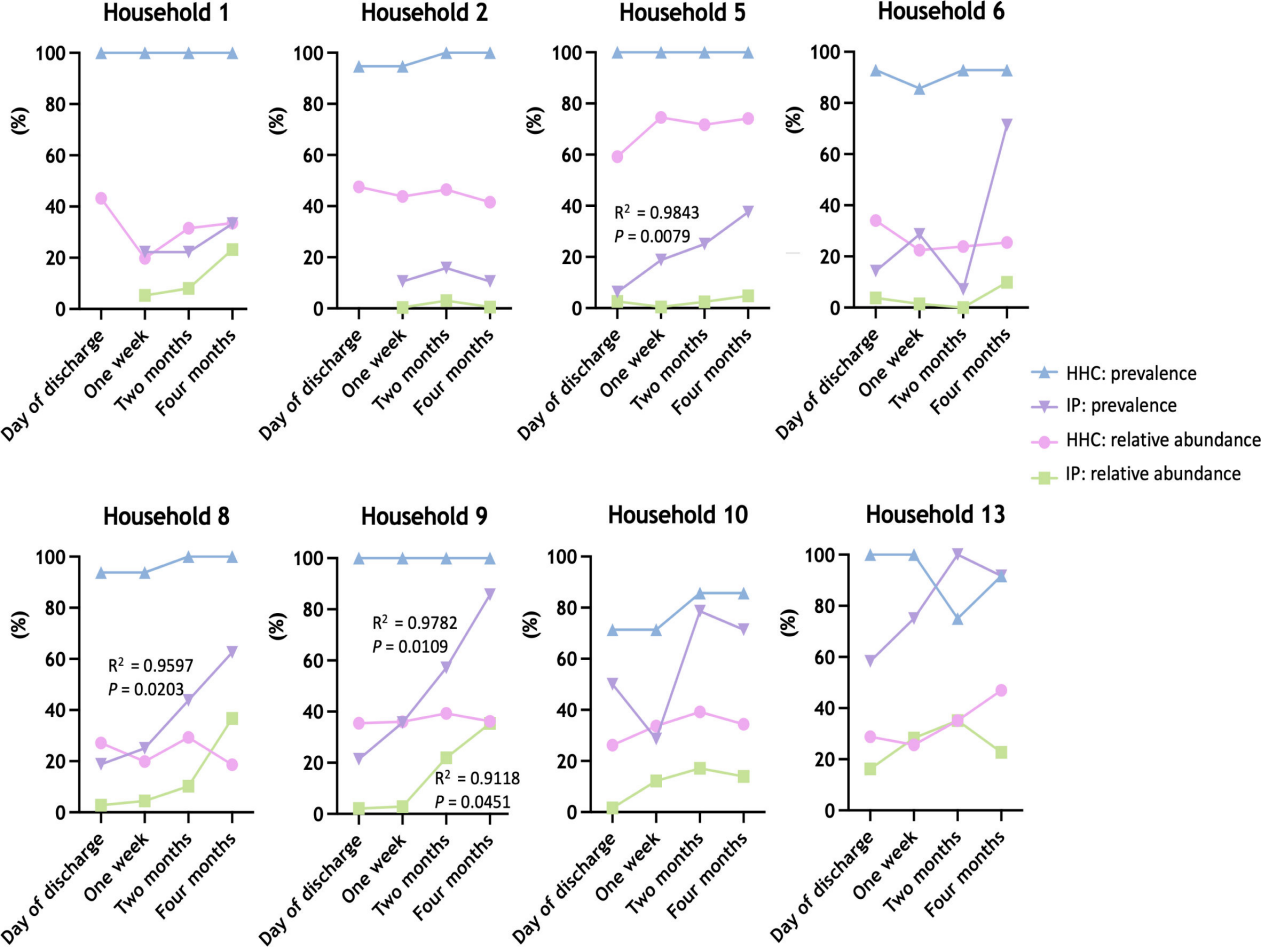
Prevalence and relative abundance of health-associated taxa (%) for each household and time point among household contacts (HHC) and index patients (IP) according to Gupta et al. (22) and Gacesa et al. (23). R^2^ values are depicted for *P* < 0.05.

To further explore whether we could identify signals of microbiome recovery in the index patients, we determined for each participant time point the presence of health-associated taxa as described by Gupta et al. and Gacesa et al. (22, 23) (Tables S3 through S10). Among all samples, in total, 22 taxa with a health association (22, 23) were found, including taxa that were present in all household contacts but variably present in index patients like *Ruminococcus* and *Subdoligranulum* or taxa-like *Paraprevotella* that was only identified in four households (households 5, 6, 8, and 10) (Tables S3 through S10). Analysis of the prevalence and relative abundance of health-associated taxa confirmed the lack of microbiome recovery in the index patients from households 1, 2, 5, and 6, where the relative abundance of health-associated taxa remained below 20% at all time points (Fig. 5; Tables S11 and S12). However, a significant (*P =* 0.0079) increase over time in prevalence of health-associated taxa could be observed for the index patient from household 5, suggesting the initiation of microbiome recovery (Fig. 5; Table S11). Also, for index patients 8, 9, 10, and 13, the prevalence and relative abundance of health-associated taxa confirmed the shift in microbiome toward their respective household contacts as observed in the PCA plot, while both revealed an over time increase, which was significant for index patient 8 for prevalence (*P =* 0.0203) and index patient 9 for both prevalence and relative abundance (*P =* 0.0109 and *P =* 0.0451, respectively) (Fig. 5; Tables S11 and S12).

## DISCUSSION

This study is a follow-up of the study by Riccio et al. which determined the rates of household acquisition and transmission of an ESBL-PE- or ESBL-positive index patient during 4 months after hospital discharge (8). Here, we used fecal samples from that study collected at the University Medical Center Utrecht to investigate gut microbiome dynamics of index patients colonized with ESBL-Ec or ESBL-Kp and their household contacts up to 4 months after hospital discharge of the index patient.

In this study, we observed large shifts in gut microbiome composition in the index patients over the different sampling moments. Beta diversity analysis using PCA and PCoA ordination of samples revealed that, at the day of discharge, the microbiome composition of index patients was very dissimilar from the household contacts reflecting a dysbiosis status at the end of hospitalization. In some of the index patients, this dissimilarity lasted throughout the 4-month follow-up period. Except for three index patients (from households 5, 8, and 9), all index patients received antibiotics prior to one or more of the sample time points. It is well recognized that antibiotic treatment results in dysbiosis and reduced diversity of the gut microbiome, which may take months to years to return to the state of pretreatment (25 – 28).

The link between dysbiosis and antibiotic treatment is reflected in the PCA ordination plots of index patients from households 1, 2, and 6 that received antibiotics throughout the study period, which revealed that the microbiome composition in these index patients remained dissimilar from the microbiome composition of their household contacts, which suggests that the microbiomes of these index patients did not recover. In contrast, the index patients from households 8, 9, and 10 only received antibiotics prior to the first time point and, in these patients, the microbiome composition converged to that of their household contacts, suggesting gradual recovery of the microbiome. The microbiome composition of index patient 13 also became less dissimilar to that of its household contacts even though this patient received antibiotics during three of the four sampling time points including prior to the last sampling time point. However, despite the fact that, in some of the index patients, there was limited recovery of the microbiome, we observed an overall significant trend in decrease of Bray-Curtis dissimilarities between household contacts and index patients over time.

It is tempting to link taxa to a health or non-health status of the gut microbiome and indeed there are some recent papers where this has been studied (22, 23). Gupta et al. introduced a Gut Microbiome Health Index which can be used to predict disease presence/absence based on an integrative analysis of ~5,000 human stool metagenomes from 34 published studies across healthy condition and 12 from different non-healthy conditions. This analysis identified seven species that were linked to health and 43 species, which were scarcely found in healthy conditions but more prevalent in disease conditions (22). Gacesa et al. profiled bacterial composition in the gut microbiome from ~8,000 Dutch individuals and did a similar categorization in health and disease status, which was largely consistent with the study by Gupta et al. (23). In our study, we focused on health-associated taxa to uncover signs of microbiome recovery. This analysis revealed a confirmation of the PCA plots. For the index patient of households 1, 5, and 6, the first signals for microbiome recovery could be observed despite a very dissimilar microbiome after 4 months. However, for index patients from households 8, 9, 10, and 13, the observed microbiome shift toward household contacts was also reflected in the prevalence and relative abundance of health-associated taxa. A limitation of this analysis is that we determined the health-associated taxa at genus level, while Gupta et al. and Gacesa et al. performed the categorization of health-associated taxa at species level, and therefore our findings are only indications of the possible species present on these genera.

In a recent review, the gut microbiome stability and resilience in relation to the interindividual response to perturbations like antibiotic treatment was studied (29). The authors concluded that an increasing number of studies has evidenced interindividual variability in extent and direction of response to diet and perturbations, which has been attributed to the unique characteristics of each individual’s microbiome (29). This conclusion is confirmed by the findings in our study, e.g., the index patient from household 13 received antibiotics at three different time points but showed signs of recovery, while in contrast the index patient from household 15 did not receive antibiotics but showed only small signals of recovery.

No important changes were observed in the gut microbiome composition of household contacts. This indicates that the gut microbiome composition of household contacts was stable over time, irrespective of whether household contacts were ESBL-PE culture positive (5/8) or not. The low relative abundance of *Escherichia-Shigella* specific reads in ESBL-Ec culture positive household contacts suggest that, in these household contacts, ESBL-Ec was only present in low numbers. In contrast, in none of the samples obtained from household contacts that were culture positive for ESBL-Kp, *Klebsiella* reads were found, which suggests that, in these individuals, ESBL-Kp was present in very low abundance, below the 16S rRNA detection limit. Overall, we can conclude that, in none of the household contacts, an increase of *Escherichia-Shigella* or *Klebsiella* reads was observed during the fourth month of follow-up nor a change in microbiome composition suggesting that a healthy microbiome is able to prevent outgrowth of ESBL-PE, a phenomenon known as colonization resistance (30, 31). Recently, Ducarmon et al. studied the potential role of the gut microbiome in controlling colonization of ESBL-Ec (10). In that study, using metagenomic sequencing of stool samples from ESBL-negative and ESBL-positive healthy individuals in a matched case-control study, no differences in diversity parameters or in relative abundance were observed between the two groups. In addition, in individuals carrying ESBL-Ec only, a very low abundance of *E. coli* was observed, which is similar to our observation. However, based on these results, the authors draw a different conclusion and hypothesized that colonization resistance against ESBL-Ec might not be that relevant as compared to enteropathogens but that longitudinal studies are necessary to confirm this. A possible explanation for this different conclusion by Ducarmon et al*.*, while the observations in their study and our study were similar, is that we consider colonization resistance the ability of the healthy microbiota to prevent expansion of potential pathogens (32), like ESBL-PE, but not complete prevention of low-level colonization by these potential pathogens.

A limitation to this study is the unavailability of a pre-treatment feces sample from the index patients and the small sample size. It is therefore not possible to directly determine the effect of the different antibiotic classes on the microbiome composition and perform in-depth differential abundance analysis of taxa.

To our knowledge, this is the first paper that studied the gut microbiome dynamics in eight index patients colonized with ESBL-PE after hospital discharge and the impact of exposure to this index patient on the gut microbiome dynamics of their household contacts. We showed that the microbiome composition from index patients is different from their household contacts upon hospital discharge and that, in some of the index patients, their microbiome composition over time shifted toward the composition of their household contacts. In contrast, household contacts showed a stable microbiome composition over time irrespective of low-level ESBL-Ec or ESBL-Kp gut colonization, suggesting that, in healthy microbiomes, colonization resistance is able to prevent ESBL-PE expansion.

## Data Availability

Sequencing data have been made available on the European Nucleotide Archive under project PRJEB60866. R scripts to reproduce the analysis reported in this study can be found at https://gitlab.com/mmb-umcu/gut_microbiome_dynamics_households.

## SUPPLEMENTAL MATERIAL

Fig. S1 and S2 spectrum.01275-23-s0001.pdf Supplemental figures.

Tables S1 to S12 spectrum.01275-23-s0002.xlsx Supplemental tables.

ASM does not own the copyrights to Supplemental Material that may be linked to, or accessed through, an article. The authors have granted ASM a non-exclusive, world-wide license to publish the Supplemental Material files. Please contact the corresponding author directly for reuse.
