# Supplementary material for: Gut microbiome dynamics in index patients colonized with extended-spectrum beta-lactamase (ESBL)-producing Enterobacterales after hospital discharge and their household contacts
Source: Microbiol Spectr. 2023 Oct 27;11(6):e01275-23. doi: 10.1128/spectrum.01275-23 (PMC10714770; doi:10.1128/spectrum.01275-23)
Supplement: Fig. S1 and S2 — Supplemental figures. [file spectrum.01275-23-s0001.pdf]

Supplementary figures

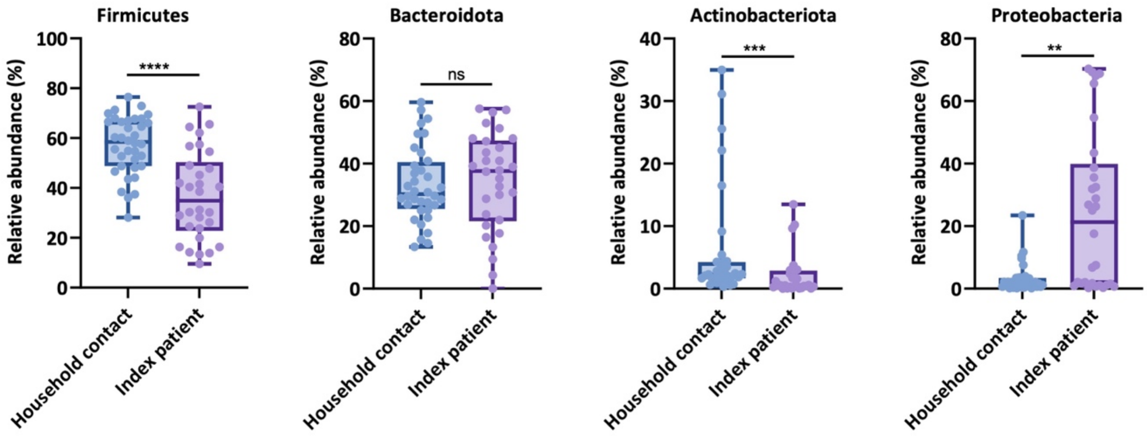

Figure S1: Distribution relative abundance for the top-4 phyla. Mann-Whitney: p-values < 0.05 are indicated by *, p-values < 0.01 are indicated by **, p-values < 0.001 are indicated by ***, p-values < 0.0001 are indicated by ****.

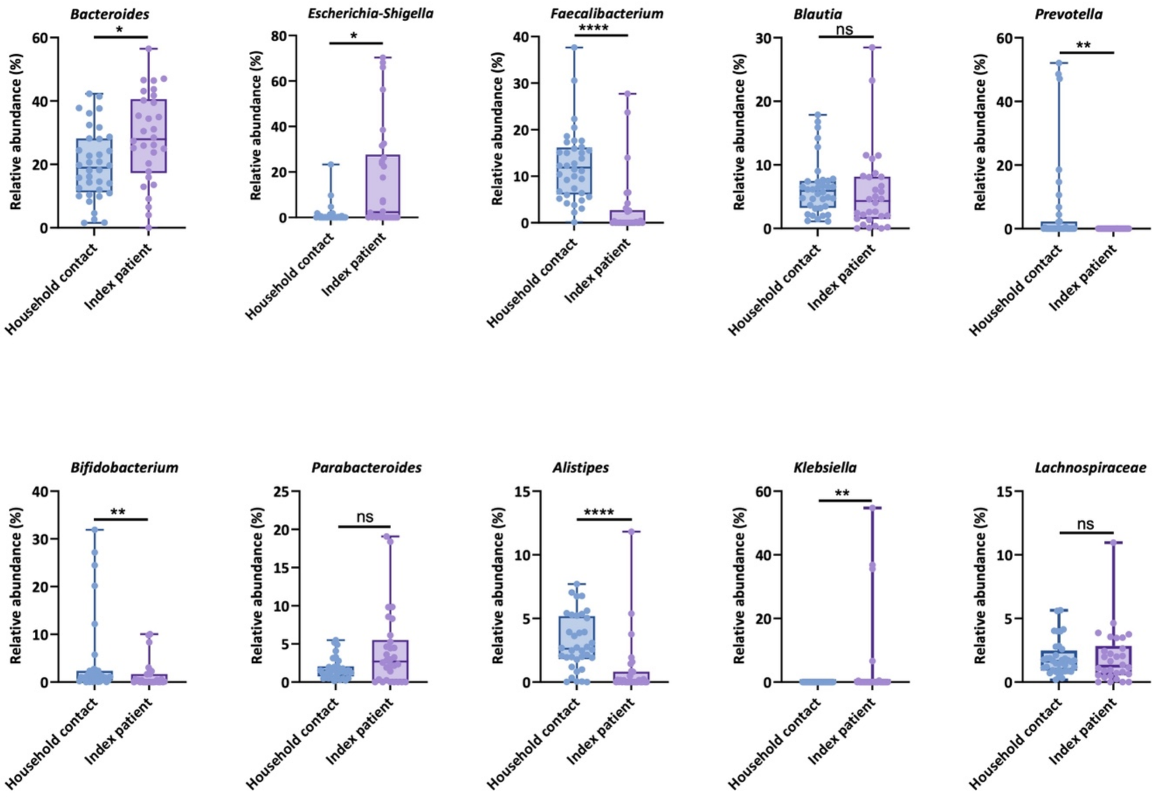

Figure S2: Distribution relative abundance for the top-10 genera. Mann-Whitney: p-values < 0.05 are indicated by *, p-values < 0.01 are indicated by **, p-values < 0.001 are indicated by ***, p-values < 0.0001 are indicated by ****.
